# Dual specificity phosphatases 10 and 16 are positive regulators of EGF-stimulated ERK activity: Indirect regulation of ERK signals by JNK/p38 selective MAPK phosphatases

**DOI:** 10.1016/j.cellsig.2011.12.021

**Published:** 2012-05

**Authors:** Ann R. Finch, Christopher J. Caunt, Rebecca M. Perrett, Krasimira Tsaneva-Atanasova, Craig A. McArdle

**Affiliations:** a Laboratories for Integrative Neuroscience and Endocrinology, School of Clinical Sciences, University of Bristol, 1 Whitson Street, Bristol, BS1 3NY, UK; b Dept. of Biology and Biochemistry, University of Bath, Claverton Down, Bath, BA2 7AY, UK; c Bristol Centre for Applied Nonlinear Mathematics, Department of Engineering Mathematics, University of Bristol, Bristol BS8 1TR, UK

**Keywords:** Epidermal growth factor, Extracellular signal activated kinase, Mitogen-activated protein kinase, MAPK phosphatase, Dual specificity phosphatase, c-Jun N-terminal kinase, p38

## Abstract

We have explored the possible role of dual specificity phosphatases (DUSPs) on acute EGF-mediated ERK signalling using high content imaging and a delayed MEK inhibition protocol to distinguish direct and indirect effects of the phosphatases on ERK activity. Using siRNAs, we were unable to find evidence that any of the MAPK phosphatases (MKPs) expressed in HeLa cells acts directly to dephosphorylate ppERK1/2 (dual phosphorylated ERKs 1 and/or 2) in the acute time-frame tested (0–14 min). Nevertheless, siRNAs against two p38/JNK MKPs (DUSPs 10 and 16) inhibited acute EGF-stimulated ERK activation. No such effect was seen for acute effects of the protein kinase C activator PDBu (phorbol 12,13 dibutyrate) on ERK activity, although effects of EGF and PDBu on ERK-dependent transcription (Egr-1 luciferase activity) were both reduced by siRNA targeting DUSPs 10 and 16. Inhibition of EGF-stimulated ERK activity by these siRNAs was reversed by pharmacological inhibition of p38 MAPK and single cell analysis revealed that the siRNAs did not influence the nuclear-cytoplasmic distribution of ppERK1/2. Thus, DUSPs 10 and 16 are positive regulators of activation, apparently acting by modulating cross-talk between the p38 and ERK pathways. A simplified mathematical model of this scenario accurately predicted the experimental data, supporting the conclusion that the major mechanism by which MKPs influence acute EGF-stimulated ERK responses is the negative regulation of p38, resulting in the positive regulation of ERK phosphorylation and activity.

## Highlights

► DUSPs could influence acute EGF effects on ERK directly or indirectly. ► The DUSPs tested had no direct negative effects on ERK signalling. ► siRNAs targeting JNK/p38-specific DUSP 10 and DUSP 16 revealed indirect modulation. ► DUSPs 10 and 16 positively modulate EGF effects by reducing p38-mediated feedback. ► DUSPs are potential therapeutic targets with effects beyond preferred substrates.

## 1 Introduction

The extracellular signal-regulated kinases (ERKs) 1 and 2 are mitogen-activated protein kinases (MAPKs) that are regulated by a diverse array of extracellular stimuli and play key roles in the control of cell fate in health and disease. They are activated by MAPK/ERK kinases (MEKs) 1 and 2 which catalyse the phosphorylation of Thr and Tyr residues in the ERK TEY activation loops [1–4]. TEY phosphorylated ERKs 1 and 2 (ppERK1/2) can phosphorylate a growing list of substrate proteins in the nucleus and cytoplasm and thereby control a large number of cellular activities [2–4]. The specificity of biological outcome from activation of ERKs is largely achieved through tight control of the duration, magnitude and localization of ERK signals. Activation of ERKs commonly causes their translocation from the cytoplasm to the nucleus, which is necessary for the transcription of many immediate early genes such as c-Fos, c-Jun and early growth response gene-1 (Egr-1) [5–9]. In fibroblasts and epithelial cells, sustained ERK activity causes expression and stabilization of immediate early gene products, culminating in G1/S transition [7–10]. This does not occur in cells where nuclear localization of ERK is prevented [11]. In contrast, transient ERK signals similarly cause the transcription of immediate early genes, but this is not sustained, and the protein products are rapidly degraded [7–11].

Epidermal growth factor (EGF) family ligands act via ErbB family tyrosine kinase receptors [12–14]. They are activated when ligand binding promotes receptor dimerization and consequent self phosphorylation on tyrosines. This facilitates recruitment of adapter proteins or enzymes that initiate signalling to a number of effectors including components of the ERK, JNK (c-Jun N-terminal kinase) and p38 (stress response kinase) MAPK pathways [12–14]. In many systems EGF causes a characteristically transient activation of ERK. Given the importance of signal duration in determining cell fate, and the importance of these receptors and effectors as therapeutic targets, there is considerable interest in the molecular mechanisms determining the kinetics of this response [2,7–9,12–18]. A large number of regulatory mechanisms have been described and these include receptor internalization [12,20] as well as ERK-mediated negative feedback at the level of SOS or Raf-1 [19]. Such mechanisms dictate the kinetics of the input for activation, and act in concert with protein phosphatases to shape ERK signals. In this regard the MAPK phosphatases (MKPs) are receiving increasing attention as physiological regulators of ERK signalling and as potential therapeutic targets. The MKP family of dual specificity phosphatases (DUSPs) can dephosphorylate and anchor ERKs, thereby influencing all three major determinants of ERK action (amplitude, kinetics and compartmentalization). The three groups of DUSPs that target MAPKs [15,21–31] are i) the nuclear-inducible MKPs (DUSP 1/MKP-1, DUSP 2/PAC1, DUSP 4/MKP-2 and DUSP 5/VH3), which all dephosphorylate ERK and, with the exception of DUSP 5, also dephosphorylate JNK and/or p38, ii) the cytoplasmic ERK MKPs (DUSP 6/MKP-3, DUSP 7/PYST2 and DUSP 9/MKP-4), which preferentially target ERK and iii) the JNK/p38 MKPs (DUSP 8/VH5, DUSP 10/MKP-5 and DUSP 16/MKP-7). Many stimuli cause an ERK-mediated increase in expression of nuclear-inducible MKPs that can dephosphorylate and anchor ERKs within the nucleus and thereby influence responses to sustained ERK activating stimuli [15]. Much less is known about the roles of DUSPs in shaping transient responses to stimuli such as EGF, where insensitivity to cycloheximide implies dependence on pre-existing (rather than induced) phosphatases [15,16]. However, we have recently screened for effects of siRNA-mediated DUSP knock-down on acute (5 min) EGF-stimulated ERK activation. We found that knock-down of individual nuclear-inducible DUSPs had no effect whereas EGF-stimulated ERK phosphorylation was reduced by siRNAs targeting DUSPs 3, 9,10 and 16 [15]. The DUSP 16 knock-down data are consistent with recent work showing that transfection with DUSP 16 can increase and prolong EGF-stimulated ERK phosphorylation [32]. Although DUSP 16 is relatively inactive toward ppERKs as substrates, it can bind and scaffold ERK2. Interestingly, it was shown to scaffold and retain ppERK1/2 in the cytoplasm of COS-7 cells, so that it actually inhibited effects of a protein kinase C (PKC) activator on expression of ERK target genes, as measured using AP-1- and SRE-luciferase reporters [32]. This illustrates the potential for DUSPs to act as positive or negative regulators of ERK, with their overall effects reflecting their ability to scaffold ERKs and to influence upstream feedback and cross-talk, as well by directly dephosphorylating their preferred MAPK targets.

Here, we have explored the possible role of DUSPs in shaping acute ERK responses to EGF in HeLa cells, using a high content imaging system for quantification of effects on ERK activation and nuclear-cytoplasmic distribution [15,16,33,34], and a “delayed MEK inhibition” protocol in which ERK dephosphorylation is followed after addition of a MEK inhibitor. This protocol is intended to reveal ERK inactivation kinetics in isolation (i.e. without any influence of negative feedback upstream of ERK or positive feed-forward activation from MEK) in order to delineate direct and indirect effects of siRNA-mediated DUSP knock-downs. We validated this approach by monitoring and modeling activation of ERK2 as compared to K52R-ERK2 (a catalytically inactive mutant used to prevent ERK-mediated negative feedback) and D319N-ERK2 (a mutant deficient in D-domain-dependent binding to proteins including DUSPs). We then used this approach to test for effects of siRNA-mediated DUSP knock-down and found no evidence for direct dephosphorylation of ERKs by DUSPs after acute activation with EGF. However, siRNAs targeting DUSPs 10 and 16 reduced the amplitude and duration of EGF-stimulated ERK phosphorylation responses, effects that were not attributable to scaffolding ERK in the cytoplasm. Instead, the effects of these siRNAs were prevented by p38 inhibition, implying that these DUSPs oppose upstream p38-mediated negative regulation of ERK. Thus, in this model the major DUSPs shaping acute EGF-mediated ERK responses are in fact JNK/p38 specific MKPs, acting by modulating cross-talk between MAPK cascades, rather than by direct effects on ERK dephosphorylation or scaffolding.

## 2 Materials and methods

### 2.1 Cell culture, transfection and transduction

HeLa cells (from the European Cell and Culture Collection, Porton Down, UK) were cultured in 10% FCS-supplemented Dulbecco's modified Eagle's medium (DMEM) without sodium pyruvate (Invitrogen Paisley, UK). For 96-well plate experiments, they were harvested by trypsinization and seeded at 3–5 × 10^3^ cells/well in Costar plain black-wall 96-well plates (Corning, Arlington, UK). They were maintained in culture for 2 days and then transferred to medium containing 0.1% FCS for 16–24 h before stimulation for various periods with EGF (Sigma-Aldrich, Gillingham, UK) or PDBu (phorbol 12, 13-dibutyrate, Sigma-Aldrich). The stimulations were terminated by washing the cells in 150 μl/well cold (< 4 °C) phosphate-buffered saline (PBS) and placing the plates on ice. The PBS was removed and the cells were fixed by adding 50 μl/well of 4% paraformaldehyde (PFA) in PBS and incubating on a rocking platform for 5 min at room temperature. The PFA solution was then removed and the cells were permeabilized by adding 50 μl/well of –20 °C methanol and incubating for 5 min at –20 °C. For some experiments, cells were treated with a 10 μM PD184352 (Enzo Life Sciences, Exeter, UK) to inhibit MEK, 10 μM SP600125 (Sigma-Aldrich) to inhibit JNK, or with 10 μM SB203580 (Sigma-Aldrich) to inhibit p38 MAPK (timing details for inhibitor addition are given in the figure legends).

For some experiments siRNA duplexes were used to knock-down endogenous ERK1 and 2, and recombinant adenovirus (Ad) were used to add-back either wild-type or mutant ERK2-green fluorescent protein (GFP) reporters. Briefly, cells were transfected using RNAiMAX reagent (Invitrogen) and the manufacturer's reverse transfection protocol was used with 2 siRNA duplexes (Qiagen, Crawley, UK) each for ERK1 and ERK2 as described [15,16,33,34]. A mixture of all 4 ERK1/2 duplexes or control siRNA against GFP (Ambion, Warrington, UK) was used in experiments at 2.5 nM total concentration. The cells were cultured for 16 h after siRNA transfection and then transduced with 1.5 × 10^6^ plaque-forming units (pfu)/ml of Ad expressing wild-type ERK2-GFP, K52R ERK2-GFP or D319N ERK2-GFP vector in DMEM with 2% FCS. These Ad were created using viral shuttle vectors constructed by subcloning a *Kpn*I–*Not*I digest of ERK2-GFP in pEGFP-N1 (a gift from Prof. Louis Luttrell, Medical University of South Carolina, Charleston, USA) into a corresponding digest of pacAd5CMV K-N pA (donated by Prof. Beverly Davidson, University of Iowa, Iowa City, USA). K52R, and D319N mutations were introduced using a QuikChange PCR-based mutagenesis kit (Stratagene, Amsterdam, NL) as described [15,16,33,34]. The Ad-containing medium was removed after 4–6 h and replaced with fresh DMEM supplemented with 0.1% FCS. The cells were then maintained for 16–24 h in this medium before stimulation. For some experiments siRNA duplexes were used to knock-down expression of endogenous DUSPs. To do so cells were transfected (as above) with 10 nM SMARTpool siRNA mixtures (Dharmacon, Cramlington, UK) targeting individual DUSPs (alone or in combination) or with a control non-targeting siRNA mixture, as described [15,16,33,34]. The cells were maintained in DMEM with 10% FCS for 24 h after siRNA transfection and then transferred to medium with 0.1% FCS for 16–14 h before stimulation, as above.

### 2.2 Immunohistochemistry and imaging

Cells were cultured in 96 well plates and after appropriate transduction and transfection, were stimulated and then fixed and permeabilized as described above. To stain endogenous ERKs, cells were blocked with 5% normal goat serum in PBS and then probed with mouse anti-ppERK1/2 monoclonal antibody (clone MAPK-YT, 1:200, Sigma-Aldrich) and rabbit anti-ERK1/2 monoclonal (clone 137F5, 1:100, Cell Signaling Technology, Danvers, MA, USA) in PBS. Alexa 488-conjugated goat anti-mouse and Alexa 546-conjugated goat anti-rabbit secondary antibodies (1:200, Invitrogen) and DAPI/PBS (600 nM) were used to visualize ppERK1/2 and ERK1/2, and to stain nuclei (respectively) as described [15,16,33,34]. For imaging ppERK2 in cells expressing ERK2-GFP, cells were counterstained with mouse anti-ppERK1/2 monoclonal (1:200) and Alexa 546-conjugated goat anti-mouse secondary (1:200) as above. Image acquisition in each well was performed on an IN Cell Analyzer 1000 (GE Healthcare, Amersham, UK) high content imaging platform, using a 10× objective and 360 nm (DAPI), 475 nm (Alexa 488 and GFP) and 535 nm (Alexa 546) excitation filters, with 460 nm, 535 nm and 620 nm emission filters, respectively, and with a 61002 trichroic mirror (GE Healthcare). Analysis of endogenous ERK1/2 and ppERK1/2 was performed using the Multi-target Analysis algorithm in the IN Cell Analyzer Workstation (GE Healthcare) using DAPI images to define nuclear perimeters and ERK1/2 or ERK2-GFP staining to define cell perimeters. These were then used as masks for quantification whole cell ppERK1/2 staining or nuclear and cytoplasmic ERK2-GFP staining. Imaging data are reported as ppERK1/2 intensity (mean fluorescence intensity per cell) or as a ratio of nuclear to cytoplasmic ERK2-GFP stain intensity (N:C ratio). For most experiments population averaged measures are reported, but for some experiments frequency–distribution curves were generated from measures in individual cells, or N:C ERK1/2 ratios were calculated for cells sorted into sub-populations according to the whole cell ppERK1/2 stain intensity as described [15,16,33,34].

### 2.3 Quantitative PCR

DUSP knock-down efficiency was tested as described [33,34] in HeLa cells that were simultaneously plated and transfected in 24-well plates (3.125 × 10^4^ cells/well) with 50 nM non-targeting control or SMARTpool siRNA mixtures targeting human DUSP 2, DUSP 10 or DUSP 16 (Dharmacon). Twenty-four hours after plating, cells were serum starved overnight and extraction of total RNA was performed 24 h later using an RNeasy kit according to the manufacturer's instructions (Qiagen). Contaminating genomic DNA was removed from columns using an additional DNase (Qiagen) digestion step. Complementary DNA was then prepared for 1 μg of each total RNA sample using a cloned avian myeloblastosis virus first-strand synthesis kit according to the manufacturer's instructions (Invitrogen). cDNAs were then quantified relative to expression of human GTPase-activating protein using the following primers: human GTPase-activating protein, 5′-GGG AAG GTG AAG GTC GGA GT-3′ and 5′-GAG TTA AAA GCA GCC CTG GTG A-3′; DUSP 2, 5′-AAA ACC AGC CGC TCC GAC-3′ and 5′-CCA GGA ACA GGT AGG GCA AG-3′; DUSP 10, 5′-GCC AGC CAC TGA CAG CAA C-3′ and 5′-TCC CAC ACT GGT GAG CTT CC-3′; and DUSP 16, 5′-TCA CTG TAC TTC TGG GTA AAC TGG AG-3′ and 5′-AAG GCT GAG AAA TGC AGG TAG G-3′. PCR primers were mixed with 50 ng of reverse transcription-PCR template and SYBR green PCR master mix (Applied Biosystems, Warrington, UK), and the comparative *C*_*T*_ method was used to detect relative expression curves on an ABI PRISM 7500 detection system (Applied Biosystems).

### 2.4 Luciferase assays

Luciferase assays were performed as described [33,34] in cells that were transfected with siRNA, transduced with Ad vectors and plated on Costar plain black-wall 96-well plates (Corning), but including Ad Egr-1 Luciferase and Ad CMV β-galactosidase reporter vectors. Following treatment with PDBu or EGF, cells were washed in ice-cold PBS, lysed and assessed for luciferase activity by chemical luminescence following the addition of luciferin substrate (Promega, Southampton, UK). β-galactosidase activity was used to correct luciferase activity for transfection efficiency, as measured following the addition of chlorophenol red-β-d-galactopyranoside substrate (Roche, Hertfordshire, UK).

### 2.5 Statistical analysis and data presentation

The cell imaging experiments and luciferase reporter experiments were performed at least 3 times, with 2–4 replicate wells for each treatment in each experiment. In order to pool data from repeat experiments, they were normalized according to internal control values as described in the figure legends. Statistical analysis was by one- or two-way ANOVA followed where appropriate by Bonferroni's post-test, accepting P < 0.05 as statistically significant. For some experiments the half-time for reduction of ppERK1/2 or ppERK2 levels was calculated by curve fitting assuming one-phase exponential decay. Statistical analysis and curve fitting were performed using GraphPad Prism version 5.01 (Graphpad Software Inc., La Jolla, CA, USA).

### 2.6 Mathematical modelling

We developed a minimal mathematical model of the ERK pathway based on previous models by introducing the positive- and negative-feedback mechanisms present in the system [9,35–37]. In this model we assume that each protein kinase (i.e. Ras, Raf, MEK, ERK, p38) has only two possible states: active and inactive, and that the total amount is conserved. The negative feedback mechanisms were assumed to be allosteric inhibition, where ppERK and p38 are considered as kinases [35]. The model equations are given in Supplemental Fig. 1.

## 3 Results and discussion

**Fig. 1:**
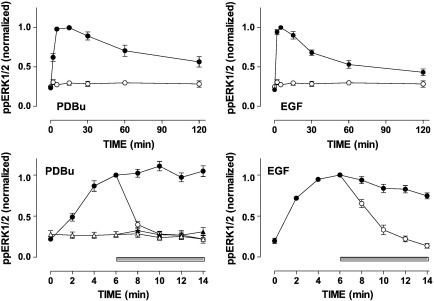
Time course of ERK activation and inactivation. Upper panels: HeLa cells were seeded in 96-well imaging plates and kept in reduced (0.1%) serum for 16 h prior to addition of 1 μM PDBu, 10 nM EGF or medium alone (open circles). After 0 or 5–120 min they were fixed and stained for endogenous ppERK1/2 and nuclei were stained with DAPI before image acquisition and analysis (as described in Materials and methods). Average whole cell ppERK1/2 measures (arbitrary fluorescence units, AFU) were calculated and normalized to internal control values (the highest observed ppERK1/2 levels, which were 385 ± 22 (n = 3) AFU and 448 ± 20 (n = 3) AFU for PDBu- and EGF-stimulated cells, respectively). The data shown are means ± SEMs (n = 3) pooled from 3 separate experiments each with triplicate or quadruplicate wells. Lower panels: HeLa cells were cultured, stimulated with EGF or PDBu (filled circles) or medium alone (open triangles), and then fixed and stained before image acquisition and analysis as above, except that a 14 min time course was used and ppERK1/2 levels were measured at 2 min intervals. For some cells, the MEK inhibitor PD184352 (10 μM) was added 6 min after the stimulus (open circles, horizontal bar) in order to follow ERK inactivation after MEK inhibition. Whole cell ppERK1/2 measures were normalized to internal control values (6 min ppERK1/2 levels for each stimulus) and the data shown are means ± SEMs (n = 3–7) pooled from 7 separate experiments each with triplicate or quadruplicate wells. Rates of ERK inactivation after MEK inhibition were estimated for the entire series of experiments (assuming exponential decay after MEK addition) and this revealed half times of 0.92 ± 0.08 min (n = 23) and 1.90 ± 0.14 min (n = 16) in the presence of PDBu and EGF, respectively.

In the first experiments we compared the time courses of ERK activation using fluorescence immunohistochemistry and automated imaging to define whole cell levels of ppERK1/2 in HeLa cells stimulated for up to 2 h with maximally effective concentrations of EGF or PDBu (Fig. 1). These stimuli caused the expected increases in ppERK1/2 levels [15,16,33,34]. Maximal responses were comparable, at 385 ± 22 (3) and 448 ± 20 (3) arbitrary fluorescence units (AFU) for PDBu and EGF, respectively. As expected, the response to EGF was rapid and transient (maximal at 5–10 min, reducing to less than 50% maximal within 30 min), as compared to the slower and more sustained effect of PDBu (maximal at 10–20 min, remaining at least 50% maximal for 60 min). We also performed shorter time-course experiments stimulating the cells for up to 14 min with EGF or PDBu and this again revealed the difference in response kinetics (maximal EGF and PDBu effects at 4–6 min or 8–14 min, respectively). When the MEK inhibitor PD184352 was added 6 min after EGF or PDBu, it caused a rapid inactivation of ERK with ppERK1/2 returning to near basal levels within 4–8 min. Curve fitting (assuming an exponential reduction in the presence of the MEK inhibitor and analysing data from the entire series of experiments) revealed that the PD184352-induced reversal of the PDBu effect was faster than reversal of the EGF effect (half-times 0.92 ± 0.08 min (n = 23) and 1.90 ± 0.14 min (n = 16), in PDBu- and EGF-stimulated cells, respectively).

**Fig. 2:**
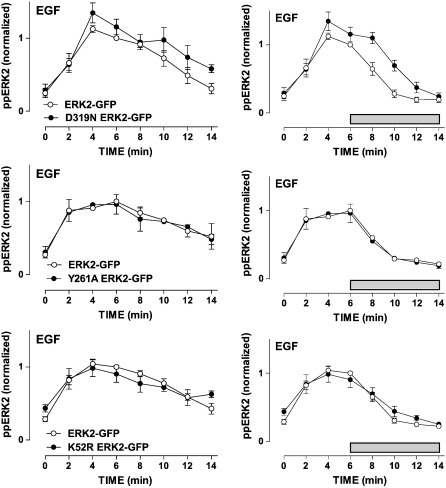
Time course of EGF-stimulated ERK activation using a knock-down and add-back model with wild-type and mutant ERK2-GFP reporters. HeLa cells were transfected with siRNAs targeting ERK1/2 and were transduced with Ad expressing wild-type ERK2-GFP, or with Ad expressing D319N ERK2-GFP, Y261A ERK2-GFP or K52R ERK2-GFP, as indicated. They were then kept in medium with 0.1% FCS for 16 h prior to stimulation (with 10 nM EGF for 0 or 2–14 min), fixation, staining and imaging as above. The left panels show time course in cells stimulated without MEK inhibitor, whereas the right panels show time courses with PD184352 (10 μM) added 6 min after the stimulus (horizontal bar). Whole cell ppERK2 measures were normalized to internal control values (6 min ppERK levels in cells with wild-type ERK2-GFP) and the data shown are means ± SEMs (n = 3–6) pooled from 6 separate experiments each with duplicate or triplicate wells. Rates of ERK inactivation after MEK inhibition were estimated (assuming exponential decay after MEK addition) and this revealed a half-time of 1.62 ± 0.12 min (n = 6) in cells expressing ERK2-GFP, that was significantly increased by the D319N mutation (3.25 ± 0.33 min (n = 4), P < 0.05) but not by the Y261A or K52R mutations (1.40 ± 0.02 min (n = 3) and 1.72 ± 0.17 min (n = 5), respectively).

The effect of the MEK inhibitor demonstrates the importance of continuous MEK activity for the observed ppERK1/2 responses and provides a method for monitoring the kinetics of ERK inactivation in isolation. That is, in normal cells ppERK1/2 responses are shaped by feed-forward activation of MEK and by negative feedback and cross-talk from other MAPK pathways upstream of MEK, as well as by direct dephosphorylation of ERK1/2, but if MEK could be completely and instantly inhibited (MEK inhibition after stimulation) ERK1/2 inactivation kinetics would reflect solely the direct dephosphorylation of ppERK1/2. A similar approach was recently used by Aoki et al. to define rate constants for ppERK1/2 dephosphorylation and this was validated by demonstrating sensitivity to serine/threonine phosphatase activity with calyculin A [38]. As an alternative approach, we used a knock-down/add-back model in which inhibitory RNAs are used to knock-down endogenous ERK1/2 and recombinant Ad are used to add-back wild-type or mutated ERK2-GFP reporters. We have previously characterized this model showing (by Western blotting, imaging and functional readouts) that expression of endogenous ERK1/2 is reduced by over 90% and that the ERK2-GFP reporters are expressed at levels comparable to control levels for endogenous ERK1/2 [15,16,33,34]. As in previous experiments, we found that the time courses for activation of ERK2-GFP by EGF and PDBu were comparable to those for activation of endogenous ERK1/2 (Figs. 1 and 2). The specificity of ERK binding to partner proteins is regulated through binding motifs including D (docking)- and DEF (docking site for ERK, FXFP)-domains. These motifs can influence interaction of ERKs with phosphatases [11,16,21,26–28] and such interactions can be perturbed by introducing specific mutations into ERK. The D319N mutation of ERK prevents association with D-domain binding partners whereas the Y261A mutation prevents DEF domain-dependent binding, and neither mutation alters the intrinsic catalytic activity of ERKs [9,39]. In contrast, the K52R mutation prevents catalytic activity. Using the knock-down/add-back protocol we obtained comparable expression levels for wild-type ERK2-GFP, D319N ERK2-GFP, Y261A ERK2-GFP and K52R ERK2-GFP (as judged by whole cell GFP measures, data not shown). The K52R mutation increased basal whole cell ppERK1/2 levels (time 0 values were 140 ± 6 (n = 5) and 206  ± 17 (n = 5) AFU in cells expressing ERK2-GFP and K52R ERK2-GFP, respectively) whereas no such effect was seen with the other mutants (Fig. 2 and Supplemental Fig. 2). EGF and PDBu caused marked increases in ppERK2 levels with all 4 reporters and responses to both stimuli were increased by the D319N mutation, but not by either of the other mutations (Fig. 2 and Supplemental Fig. 2). When the MEK inhibitor PD184352 was added after 6 min it caused a rapid decline in ppERK2 levels under all conditions. Importantly however, the half-time for the reduction in ppERK1/2 was unaltered by the K52R and Y261A mutations (P > 0.05), but was increased at least 2 fold by the D319N mutation in EGF-stimulated cells (half-time increased from 1.62 ± 0.12 min (6) to 3.25 ± 0.33 min (4), P < 0.01) and in PDBu-stimulated cells (half-time increased from 1.19  ± 0.22 min (6) to 3.32 ± 0.21 min (4), P < 0.01) (Fig. 2 and Supplemental Fig. 2). The simplest explanation for this increased half-time is that ERK2 is dephosphorylated by D-domain containing phosphatase(s) and it is well established that MKPs have N-terminal D-domains, which determine substrate specificity [8,22]. By the same logic, the Y261A mutation provides a negative control expected not to alter direct dephosphorylation of ERK2 by DUSP 1 and DUSP 4. The K52R mutation also provides a control as it is thought to increase basal ppERK2 levels by preventing ERK-dependent negative feedback and the lack of effect of this mutation on half-time for ERK inactivation (in the presence of the MEK inhibitor) is entirely consistent with this interpretation. To further explore these possibilities we developed a mathematical model (Supplemental Fig. 1), using previously described pathway architecture and rate constants [19] to describe the effects of EGF on the ERK signalling cascade in terms of a series of ordinary differential equations. We used the data in Fig. 1 to train the model parameters, simplifying ERK inactivation to a single dephosphorylation step with a rate constant of 0.42 min^− 1^. We then validated the model (model A = steps 1–4 shown in Supplemental Fig. 1) using the data from Fig. 2 and from Supplemental data Fig. 2. Consistent with the interpretation above, the model predictions closely paralleled the experimental data when we assumed a) that PD184352 causes complete and instantaneous blockade of MEK, b) that the sole effect of the D319N mutation is to increase the half-time for ERK inactivation from 1.62 to 3.25 min, and c) that the sole effect of the K52R mutation is to prevent ERK-mediated negative feedback at the level of SOS or Raf (compare Figs. 2 and Supplemental Figs. 2 and 3).

**Fig. 3:**
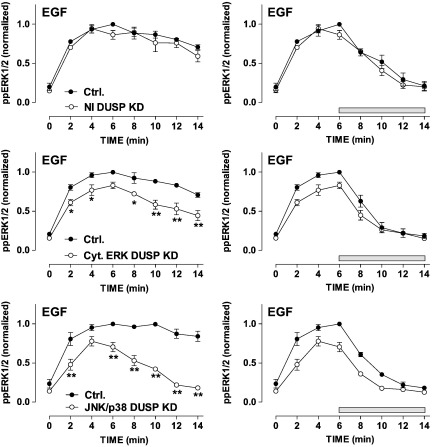
Influence of DUSP knock-down on the time course of EGF-stimulated ERK activation. HeLa cells were transfected with control siRNA (Ctrl., all panels) or with a combination of siRNAs targeting the nuclear-inducible DUSPs 1, 2, 4 and 5 (NI DUSP KD, upper panels), the cytoplasmic ERK-directed DUSPs 6, 7 and 9 (Cyt. ERK DUSP KD, middle panels) or the JNK/p38 DUSPs 10 and 16 (JNK/p38 DUSP KD, lower panels) and then kept in medium with 0.1% FCS for 16 h prior to stimulation (with 10 nM EGF for 0 or 2–14 min), fixation, staining and imaging as above. The left panels show time course in cells stimulated without MEK inhibitor, whereas the right panels show time courses with PD184352 (10 μM) added 6 min after the stimulus (horizontal bar). Whole cell ppERK1/2 measures were normalized to internal control values (6 min ppERK1/2 levels in cells with control siRNA) and the data shown are means ± SEMs (n = 3) pooled from 3 separate experiments each with duplicate or triplicate wells. *P < 0.05, **P < 0.01 for comparison of control and test siRNAs. Rates of ERK inactivation after MEK inhibition were estimated (assuming exponential decay after MEK addition). The half-time was 2.61 ± 0.30 min (n = 3) in cells with control siRNA and this was not significantly different (P > 0.05) to the half times in cells with siRNAs targeting any of the DUSP families.

Together, the mathematical and experimental approaches described above demonstrate the potential for modulation of basal and stimulated ppERK1/2 levels by direct alteration of dephosphorylation or by alteration of feedback, and the utility of delayed MEK inhibitor protocol for delineating these direct and indirect effects. Accordingly, we next used these models to further explore possible effects of DUSPs using siRNA to knock down endogenous DUSPs and automated imaging to monitor activation of endogenous ERK1/2. Combined knock-down of nuclear-inducible DUSPs 1, 2, 4 and 5 had no measurable effect on ppERK1/2 responses to EGF or PDBu either alone, or when PD184352 was added at 6 min (Fig. 3 and Supplemental Fig. 4). These data are not unexpected because induced expression of these phosphatases would not occur in the short time-frame of these experiments and because we have previously shown that individual knock-downs of these DUSPs have no measurable effect on acute (5 min) EGF-stimulated ppERK2 levels using our knock-down/add-back protocol [15]. Combined knock-down of the cytoplasmic ERK-directed DUSPs 6, 7 and 9 reduced ppERK1/2 responses to both stimuli at all time points measured in the absence of PD184352 but, importantly, did not alter the half-times for reduction in ppERK1/2 levels when PD184352 was added at 6 min (Fig. 3 and Supplemental Fig. 4). Combined knock-down of the JNK/p38 DUSPs 10 and 16 reduced the EGF effect on ppERK1/2 at all time points measured and made the response remarkably transient, returning to basal values within 14 min (Fig. 3). This effect of DUSPs 10 and 16 knock-down on response kinetics was confirmed by two-way ANOVA in which ”time”, “knock-down” and the “time–knock-down interaction” terms were all statistically significant (P < 0.01). However, it was not associated with any measurable change in the half-time for ppERK1/2 inactivation in the delayed MEK inhibition protocol. Moreover, in contrast to the situation with siRNAs targeting the cytoplasmic ERK MKPs, knock-down of JNK/p38 MKPs specifically reduced responses to EGF without any effect on the PDBu responses (compare Fig. 3 with Supplemental Fig. 4). We also confirmed effectiveness of the knock-down, as combined siRNAs targeting DUSPs 10 and 16 reduced transcription of these targets (each by 80-90% as judged by qPCR) without reducing DUSP 2 expression (not shown).

**Fig. 4:**
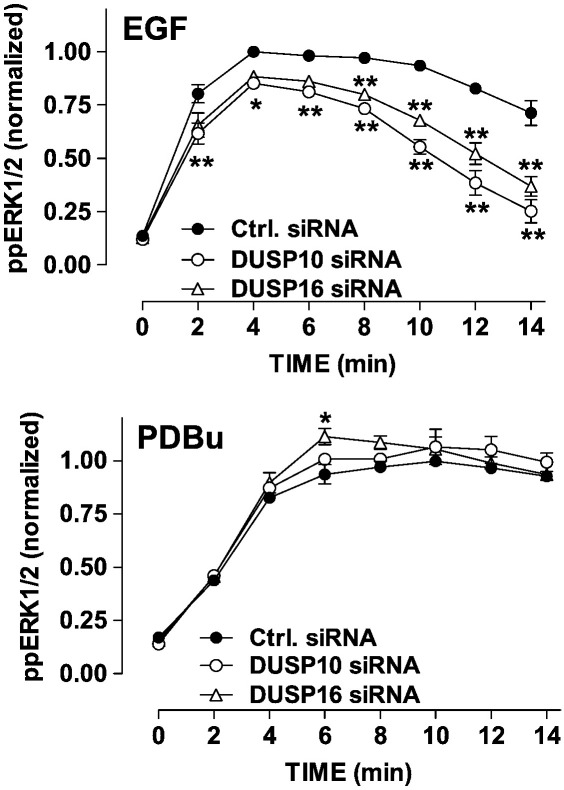
Influence of JNK/p38 DUSP knock-down on the time course of EGF-stimulated ERK activation. HeLa cells were transfected with control siRNA (Ctrl, all panels) or with siRNAs targeting either DUSP 10 or DUSP 16. They were then kept in medium with 0.1% FCS for 16 h prior to stimulation (with 10 nM EGF or 1 μM PDBu for 0 or 2–14 min), fixation, staining and imaging as above. Whole cell ppERK1/2 measures were normalized to internal control values (6 min ppERK1/2 levels in cells with control siRNA) and the data shown are means ± SEMs (n = 3) pooled from 3 separate experiments each with duplicate or triplicate wells. *P < 0.05, **P < 0.01 for comparison of control and test siRNAs.

**Fig. 5:**
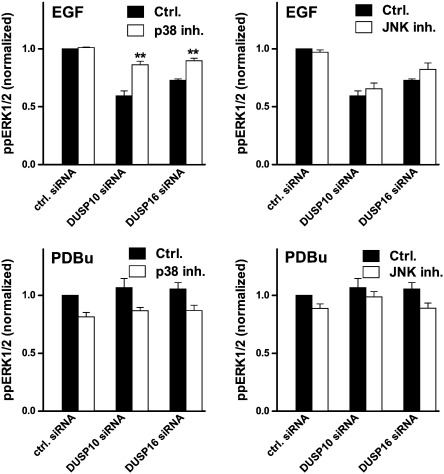
Influence of JNK and p38 inhibitors on modulation of ERK activation by DUSP knock-down. HeLa cells were transfected with control siRNA (ctrl.) or with siRNAs targeting either DUSP 10 or DUSP 16 as indicated. They were then kept in medium with 0.1% FCS for 16 h and stimulated for 10 min with 10 nM EGF (upper panels) or with 1 μM PDBu (lower panels), before being fixed, stained and imaged as above. To determine inhibitor effects, cells were pre-treated for 30 min with 10 μM SB203580 p38 inhibitor (p38 inh., left panels), with 10 μM SP600125 JNK inhibitor (JNK inh., right panels) or with medium alone (Ctrl, all panels) before addition of the stimulus. Average whole cell ppERK1/2 measures were calculated and normalized to internal control values (ppERK1/2 levels in EGF- or PDBu-stimulated cells with control siRNA and no inhibitor). The data shown are means ± SEMs (n = 3) pooled from 3 separate experiments each with triplicate wells. Two-way ANOVA of the data in A revealed that DUSP siRNA, p38 inhibitor and the siRNA/inhibitor interaction were all significant variables (P < 0.01) and post-hoc tests revealed that p38 inhibition increased ppERK1/2 levels in the presence of either siRNA (**, P < 0.01) but not in its absence. ANOVA for the data in B revealed significance only for the DUSP siRNA effect (P < 0.01) and the post-hoc tests showed no significant effects of JNK inhibition. ANOVAs performed for the PDBu-treated cells (C and D) revealed each of the inhibitors to be significant variables (P < 0.01 for p38 inhibition and P < 0.05 for JNK inhibition) but the siRNA and interaction terms were not significant, nor was statistical significance revealed by the post-hoc tests.

Exploring effects on ERK response kinetics further, we used siRNA to knock-down DUSP 10 or 16 individually. Again, qPCR revealed target knock-down efficiency of 80–90% (not shown) and we found that both manipulations inhibited EGF-stimulated ppERK1/2 responses at all time points measured (Fig. 4). The effect of the DUSP 10 siRNA was more pronounced than that of the DUSP 16 siRNA and neither had any measurable effect on responses to PDBu (Fig. 4). We also used pharmacological inhibition to test for dependence of these inhibitory effects on p38 or JNK. As shown (Fig. 5), siRNAs targeting DUSPs 10 and 16 significantly reduced ppERK1/2 responses elicited by 10 min stimulation with EGF and this inhibitory effect was partially prevented by p38 inhibition (with SB203580) but not by JNK inhibition (with SP600125). In parallel experiments PDBu-stimulated ppERK1/2 responses were uninfluenced by the siRNAs (Fig. 5). Moreover, although there was an overall tendency for each of the inhibitors to reduce ppERK1/2 levels alone, neither of them significantly influenced the PDBu-stimulated ppERK1/2 levels in the presence of the control or test siRNAs (Fig. 5).

The data above suggest that the JNK/p38 MKPs have no direct effect on ERK activity in cells acutely stimulated with EGF (because they have no effect on inactivation kinetics in the presence of the MEK inhibitor) but instead have an indirect positive regulatory effect that is overcome by siRNA-mediated knock-down. In these cells, PKC is thought to feed into the ERK pathway by activation of Raf, rather than by transactivation of EGF receptors as in some models [15,16,33,34] so the observed ligand specificity and reversal by p38 inhibition implies a) that the positive regulatory effect is exerted at or distal to the EGF receptor but also upstream of Raf and b) is p38 mediated. We further explored this possibility by mathematical modeling, extending model A (above) to incorporate parallel activation of two MAPK pathways (ERK and p38) and negative feedback of p38 at upstream points in the EGF receptor/ERK pathway (model B = steps 1–6 in Supplemental Fig. 1). The model prediction is that p38 targeted DUSPs will reduce p38 activity, thereby reducing the negative feedback and enhancing the ERK response. Conversely, the model predicts that knock-down of the p38 DUSP will increase the negative feedback and thereby reduce the ERK response (Supplemental Fig. 5). Clearly mathematical modeling alone cannot prove or disprove this signal architecture but it is consistent with experimental data showing that EGF causes parallel activation of ERK and p38 in some models [40–42], and that p38 activation can accelerate EGF receptor internalisation [42] providing a potential mechanism for the upstream negative feedback. It is also entirely consistent with the knock-down data and inhibitor experiments shown here (Figs. 3–5).

**Fig. 6:**
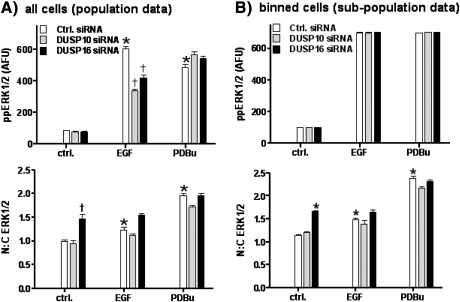
Relationships between ERK phosphorylation and nuclear translocation. Panel A: HeLa cells were transfected with control siRNA or with siRNAs targeting either DUSP 10 or DUSP 16, as indicated. They were then kept in medium with 0.1% FCS for 16 h prior to stimulation EGF (10 nM) PDBu (1 μM) or medium alone (ctrl.) for 10 min. They were then fixed, stained and imaged as above, except that the cells were stained for total ERK1/2 as well as ppERK1/2 and DAPI, and image analysis was used to calculate not only whole cell ppERK1/2 levels, but also average nuclear and cytoplasmic ERK1/2 staining intensity, so that the nuclear:cytoplasmic ratio (N:C ERK1/2) could be calculated. The data shown are means ± SEMs. * P < 0.05 compared to control cells without stimulus or DUSP siRNA. † P < 0.05 compared to the stimulus matched control siRNA group (open bars). Panel B: cells from the same experiment were binned (mathematically) according to whole cell ppERK1/2 levels, to generate sub-populations of control cells in which ppERK1/2 was 80–120 AFU, and EGF- or PDBu-stimulated cells in which ppERK1/2 was 680–720 AFU. The N:C ERK1/2 ratio was then calculated for these binned cell sub-populations. The data shown are means ± SEMs. * P < 0.05 compared to control cells without stimulus or DUSP siRNA. † P < 0.05 compared to matched control siRNA group (open bars). Note that knock-down of DUSP 10 or 16 has no measurable effect on ppERK1/2 levels (upper right) because the data are binned, yet EGF, PDBu and DUSP 16 siRNA all increased N:C ERK1/2 in these ppERK1/2 matched populations.

Our data also support a recent study showing that over-expression of DUSP 16 converts a modest and transient EGF-stimulated ppERK1/2 response to a more pronounced and sustained response in COS-7 cells [32], yet the mechanism proposed for the latter effect differs markedly from the one suggested here. In COS-7 cells, over-expression of DUSP 16 enhanced ppERK1/2 responses to activation with PDBu as well as EGF [32], acting by scaffolding and retaining ppERK1/2 within the cytoplasm and thereby preventing ERK-mediated transcriptional responses. Having used an automated cell imaging readout for ERK1/2 activation we have accumulated measures of nuclear and cytoplasmic ERK1/2 and ppERK1/2 for many thousands of individual cells and therefore used this data to explore possible effects of DUSP knock-downs on ERK1/2 distribution in our HeLa cell model (Supplemental Figs. 6 and 7). We first used population averaged responses and, as expected, these revealed pronounced and comparable stimulatory effects of EGF and PDBu on whole cell ppERK1/2 levels, as well as inhibition of the EGF effect (but not the PDBu effect) by siRNAs targeting DUSP 10 or 16 (Fig. 6A, see also Fig. 4). As with earlier work showing stimulus-induced translocation of ERK from the cytoplasm to the nucleus, we found that EGF and PDBu both increased the N:C ERK1/2 ratio and, as expected, the PDBu effect was more pronounced than that of EGF (Fig. 6A, see also Refs. [15,16,33,34]). DUSP 10 siRNA had no measurable effect on N:C ERK1/2 under any condition, (arguing against a scaffolding role) whereas knock-down of DUSP 16 caused a pronounced increase in N:C ERK1/2 in control cells (Fig. 6A). This occurred without any measurable change in ppERK1/2 levels and therefore suggests that endogenous DUSP 16 does indeed scaffold and retain ERK1/2 in the cytoplasm of unstimulated cells. However, the effects of DUSP 16 siRNA and stimulation (with EGF or PDBu) were not additive and DUSP 16 siRNA failed to cause any measurable change in N:C ERK1/2 in the presence of EGF or PDBu, arguing against a scaffolding function in the stimulated cells (Fig. 6A).

We further explored a possible scaffolding effect in sub-populations of cells binned according to ppERK1/2 levels as described [33]. We first examined frequency–distribution plots for whole cell ppERK1/2 and N:C ERK1/2 levels in control and PDBu-stimulated cells treated with control siRNA or DUSP 16 siRNA (Supplemental Fig. 7) in order to select narrow bins of ppERK1/2 levels representative of control and stimulated cells (80–120 and 680–720 AFU, respectively). We then calculated N:C ERK1/2 values in these sub-populations. Here, the idea is that comparison of test and control cells with matched ppERK1/2 levels reveals effects on distribution that are not due solely due to TEY phosphorylation of ERK. For example, we have shown that sustained stimulation with PDBu causes a pronounced increase in N:C ERK1/2 in sub-populations matched for ppERK1/2, an effect that likely reflects the ability of PDBu to increase expression of nuclear-inducible MKP scaffolds [33]. Taking a similar approach to explore any possible scaffolding effect of DUSPs 10 and 16, we found that ppERK1/2 levels in the binned cells were (inevitably) higher in the stimulated cells than the controls and importantly, were not measurably influenced by the siRNAs (Fig. 6B upper panel). The N:C ERK1/2 values were higher in EGF- or PDBu-stimulated cells than in the control cells (largely because of the higher ppERK1/2 values) and the N:C ERK1/2 values were higher in PDBu-stimulated cells than after EGF stimulation (Fig. 6B), consistent with earlier work showing that PDBu causes a pronounced nuclear accumulation of ERK that is not entirely attributable to TEY phosphorylation of ERK1/2 [33]. siRNA targeting DUSP 16 caused a pronounced increase in N:C ERK1/2 (under conditions in which it had no effect on whole cell ppERK1/2), confirming the scaffolding effect of endogenous DUSP 16 in control cells. However, knock-down of DUSP 10 had no effect on N:C ERK2-GFP under any condition, and knock-down of DUSP 16 had no measurable effect on N:C ERK1/2 in the presence of EGF or PDBu (Fig. 6B). Thus our data demonstrate a TEY phosphorylation unattributable effect of DUSP 16 siRNA (that most likely reflects a cytoplasmic scaffolding effect of DUSP 16), but only in control cells. No such effect was seen in the presence of EGF or PDBu, arguing against a scaffolding function in stimulated HeLa cells.

**Fig. 7:**
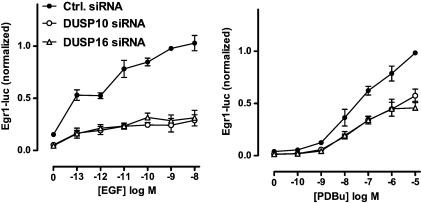
Influence of DUSPs 10 and 16 knock-down on ERK-mediated transcription. HeLa cells were transfected with control siRNA or with siRNAs targeting either DUSP 10 or DUSP 16, and incubated with Ad Egr-1 Luciferase or Ad CMV β-galactosidase reporter vectors. After culture, they were stimulated for 2 h with the indicated concentrations of EGF (left panel) or PDBu (right panel), washed, lysed and assessed for luciferase activity as described in Materials and methods. Luciferase activity was normalized to the internal control maximal responses for EGF and PDBu respectively) and are pooled from 3 separate experiments, each with 3 replicates (mean ± SEM, n = 3). Two-way ANOVA and Bonferroni post-test revealed significant inhibition (P < 0.01) at all EGF concentrations with both DUSP 10 siRNA and DUSP 16 siRNA, although neither has a significant effect in the control cells without EGF. Similarly, DUSP 10 siRNA significantly reduced responses to PDBu at 10^− 8^ to 10^− 5^ M (P < 0.05 or P < 0.01). DUSP 16 siRNA also significantly reduced responses to PDBu at 10^− 8^ to 10^− 5^ M (P < 0.01) and again, neither siRNA had a significant effect in control cells without PDBu.

We also tested for possible effects of siRNAs targeting DUSPs 10 and 16 on ERK-mediated transcription using an Egr-1 luciferase (luc) reporter. As shown (Fig. 7), stimulation for 2 h with EGF and PDBu caused dose-dependent increases in Egr-1-luc activity (see also Refs. [15,33,34]). Knock-down of either DUSP reduced the transcriptional effects of EGF, as anticipated from the effect of these knock-downs on ppERK1/2 (Fig. 4). Specificity is indicated by the fact that no significant inhibition was seen after knock-down of DUSP 2 (not shown). Surprisingly, siRNAs targeting DUSPs 10 or 16 also inhibited Egr-1-luc responses to PDBu (Fig. 7) in spite of having no effect on ppERK1/2 responses in cells stimulated 0–14 min (Fig. 4). However, this discrepancy may simply reflect differences in time courses, as we have previously shown that knock-down of DUSP 10 or 16 does reduce PDBu effects on ppERK1/2 at 2–4 h [15]. We attempted to address this by varying the stimulation period in the transcription assays but qualitatively similar data were obtained with 1, 2 and 4 h stimulation (not shown) and shorter incubation periods were considered impractical. We speculate that knock-down of these DUSPs would differentially modulate acute ERK-mediated effects (i.e. phosphorylation and regulation of pre-existing cytoplasmic targets) but not chronic effects (i.e. those dependent upon protein neosynthesis). Most importantly, however, DUSPs 10 and 16 knock-down effects reveal these DUSPs as positive regulators of EGF and ERK-driven transcription, as opposed to the negative regulatory role of DUSP 16 seen in COS-7 cells [32]. The reason for this difference is unknown although it could clearly relate to the use of different cell types (HeLa versus COS-7) in which the signal network architecture places different reliance on scaffolding versus p38-mediated cross-talk to shape ERK signals. Another possibility is that a scaffolding function of DUSP 16 in stimulated cells is negligible at physiological expression levels and is therefore revealed by over-expression [32] but not by knock-down (Fig. 6).

## 4 Conclusions

**Fig. 8:**
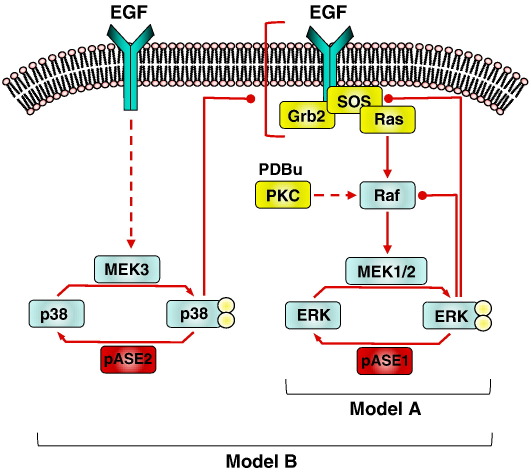
Schematic representation of acute ERK regulation pathways. Model A indicates the initial pathway with ppERK1/2 mediated negative feedback but without ppp38-mediated cross-talk (equations 1–4 in Supplemental Fig. 1). Model B incorporates negative feedback from ppp38 (equations 1–6 in Supplemental Fig. 1)). Our data reveal no role for direct DUSP-mediated inactivation of ppERK (by phosphatase (pASE) 1 in model A) in shaping ERK responses. Instead, they suggest that acute EGF effects on ERK are subject to upstream ppp38-mediated negative feedback that is enhanced by knock-down of DUSPs 10 and/or 16 (pASE 2 in model B). Thus, the major effect of DUSPs on acute EGF effects in this model appears to be exerted by modulation of cross-talk rather than by direct dephosphorylation of ppERK1/2.

We have explored the possible role of DUSPs on acute EGF-mediated ERK signalling using high content imaging and a delayed MEK inhibition protocol to distinguish direct and indirect effects on ERK activity. We were unable to find evidence that any of the MKPs expressed in HeLa cells act directly to dephosphorylate ppERK1/2 in the acute time-frame tested. Instead, we find that two p38/JNK MKPs (DUSPs 10 and 16) are positive regulators of acute EGF-stimulated ERK activation, apparently acting by modulating cross-talk between the p38 and ERK pathways. In this model (shown as a cartoon in Fig. 8 and mathematically as equations 1–6 in Supplemental Fig. 1) the major mechanism by which MKPs influence acute EGF-stimulated ERK responses is the negative regulation of p38, resulting in the positive regulation of ERK phosphorylation and activity.

## Abbreviations

PKC: protein kinase C
ERK: extracellular signal-regulated kinase
ppERK: (prefixed pp to denote dual phosphorylation in the TEY domain, suffixed 1 or 2 to denote ERK1 or ERK2, or ERK1/2 to mean ERKs 1 and/or 2)
MAPK: mitogen-activated protein kinase
MEK: MAPK/ERK kinase
EGF: epidermal growth factor
MKP: MAPK phosphatase
DUSP: dual specificity phosphatase
JNK: c-Jun N-terminal kinase
D-: docking
siRNA: short inhibitory RNA
GFP: (enhanced) green fluorescent protein
KD: knock-down
PDBu: phorbol 12,13 dibutyrate
PBS: phosphate-buffered saline
qPCR: quantitative polymerase chain reaction
DAPI: 4′-6-Diamidino-2-phenylindole
N:C: nuclear:cytoplasmic
ctrl: control
inh.: Inhibitor
AFU: arbitrary fluorescence units
luc: luciferase
pASE: protein phosphatase
DMEM: Dulbecco's modified Eagle's medium
PFA: paraformaldehyde
