# Supplementary material for: Dual specificity phosphatases 10 and 16 are positive regulators of EGF-stimulated ERK activity: Indirect regulation of ERK signals by JNK/p38 selective MAPK phosphatases
Source: Cell Signal. 2012 May;24(5):1002–11. doi: 10.1016/j.cellsig.2011.12.021 (PMC3314959; doi:10.1016/j.cellsig.2011.12.021)
Supplement: Supplementary file 1 — Supplementary materials [file mmc1.pdf]

Supplemental Figure legends.

Supplemental figure 1. A minimal mathematical model for the ERK pathway. We generated a minimal mathematical model (based on previous models) by introducing the positive- and negative-feedback mechanisms present in the system (9,35-37). In this model we assume that each protein kinase (i.e. Ras, Raf, MEK, ERK, p38) has only two possible states: active and inactive, and that the total amount is conserved. The negative feedback mechanisms were assumed to be allosteric inhibition, where ppERK and p38 are considered as kinases (35). The basic equations are given above where [*Ras*] and [*Raf*] denote the activated forms of Ras and Raf, respectively; [*ppMEK*] and [*ppERK*] denote the activated forms of MEK and ERK, respectively; and [*ppMEK3*] and [*ppp38*] denote the activated forms of MEK3 and p38, respectively. The subscript *t* of each protein kinase denotes the total amount of protein that is assumed constant. Finally *s(t)* denotes the activating stimulation. The key parameters are given in the legend to Supplemental figures 3 and 5.

Supplemental figure 2. Time course of EGF-stimulated ERK activation using a knock-down and add-back model with wild-type and mutant ERK2-GFP reporters. HeLa cells were transfected with siRNAs targeting ERK1/2 and were transduced with Ad expressing wild-type ERK2-GFP, D319N ERK2-GFP, Y261A ERK2-GFP or K52R ERK2-GFP, as indicated. They were then kept in medium with 0.1% FCS for 16 hours prior to stimulation (with 1 μM PDBu for 0 or 2-14 min), fixation and staining as described in the Materials and Methods. The left panels show time-course in cells stimulated without MEK inhibitor, whereas the right panels show time-courses with PD184352 (10 μM) added 6 min after the stimulus (horizontal bar). Whole cell ppERK2 measures were normalized to internal control values (6 min ppERK levels in cells with wild-type ERK2-GFP) and the data shown are means ± SEMs (n=2-6) pooled from 6 separate experiments each with duplicate or triplicate wells. Rates of ERK inactivation after MEK inhibition were estimated (assuming exponential decay after MEK addition) and this revealed a half-time of 1.19 ± 0.12 min (n=6) in cells expressing ERK2-GFP, that was significantly increased by the D319N mutation (3.32 ± 0.321 min (n=4), $P<0.05$) but not by the Y261A or K52R mutations.

Supplemental figure 3. Simulated time course of EGF-stimulated ERK activation using the initial model (model A = equations (1) – (4) without negative feedback term due to inhibition by [*ppp*38]). The left panels show normalized [*ppERK*] time-course in the model without MEK inhibitor, whereas the right panels show time-courses with K_{cat_3} set to zero 6 min after the stimulation with *s(t)* = 10 nM (horizontal bar). Model parameter values used in this simulations are: $K_{cat\_1} = 50$ μM^{-1}min^{-1}, $K_{cat\_2} = 5000$ μM^{-1}min^{-1}, $K_{cat\_3} = 5000$ μM^{-1}min^{-1}, $K_{cat\_4} = 500$ μM^{-1}min^{-1}, $K_{m\_1} = 0.001$ μM, $K_{m\_2} = 90$ μM, $K_{m\_3} = 11$ μM, $K_{m\_4} = 136.88$ μM, $d_{Ras} = 0.05$ min^{-1}, $d_{Ras} = 0.05$ min^{-1}, $d_{Raf} = 0.5$ min^{-1}, $d_{ppMEK} = 0.9$ min^{-1}, $d_{ppERK} = 0.42$ min^{-1}, $K_{i\_\mathrm{erk}} = 0.01$ μM, $K_{i\_\mathrm{raf}} = 0.1$ μM, $Ras_t = 0.35$ μM, $Raf_t = 0.12$ μM, $MEK_t = 0.36$ μM, $ERK_t = 0.7$ μM. The rates of ERK inactivation after MEK inhibition in the model correspond to those determined experimentally a half-time of 1.62 min is equivalent to $d_{ppERK} = 0.42$ min^{-1}, and was reduced by half in order to simulate D319N mutation, i.e. $d_{ppERK} = 0.21$ min^{-1} that is equivalent to a half-time of 3.25 min. In the case of K52R mutations we used $d_{ppERK} = 0.42$ min^{-1} consistent with experimental observations (Fig. 2) and removed the negative feedback by setting $K_{i\_\mathrm{erk}} = 1$ μM, $K_{i\_\mathrm{raf}} = 1$ μM. We also varied the initial conditions in order to reflect the difference in steady state caused by the presence of the K52R mutation. The decay rates have been calculated using the following relationship $d = \log 2/t_{1/2}$.

Supplemental figure 4. Influence of DUSP knock-down on the time course of PDBu-stimulated ERK activation. HeLa cells were transfected with control siRNA (Ctrl., all panels) or with a combination of siRNAs targeting the nuclear-inducible DUSPs 1, 2, 4 and 5 (NI DUSP KD, upper panels), the cytoplasmic ERK-directed DUSPs 6, 7 and 9 (Cyt. ERK DUSP KD, middle panels) or the JNK/p38 DUSPs 10 and 16 (JNK /p38 DUSP KD, lower panels) and then kept in medium with 0.1% FCS for 16 hours prior to stimulation (with 10 nM EGF for 0 or 2-14 min), fixation, staining and imaging as above. The left panels show time-course in cells stimulated without MEK inhibitor, whereas the right panels show time-courses with the MEK inhibitor PD184352 (10 μM) added 6 min after the stimulus (horizontal bar). Whole cell ppERK1/2 measures were normalized to internal control values (6 min ppERK1/2 levels in cells with control siRNA) and the data shown are means ± SEMs (n=3) pooled from 3 separate experiments each with duplicate or triplicate wells. **P<0.01 for comparison of control and test siRNAs. Rates of ERK inactivation after MEK inhibition were estimated (assuming exponential decay after MEK addition). The half-time was 1.19 ± 0.42 min (n=3) in cells with control siRNA and this was not significantly different (P>0.05) to the half-times in cells with siRNAs targeting any of the DUSP families.

Supplemental figure 5. Simulated time course of EGF-stimulated ERK activation using the extended model (model B = equations (1) – (6)). The left panel shows normalized [*ppERK*] time-course in the model without MEK inhibitor, whereas the right panel shows time-courses with K_{cat_3} set to zero 6 min after the stimulation with $s(t)$ = 10 nM (horizontal bar). Model parameter values used in this simulations are: K_{cat_1} = 50 $\mu M^{-1}min^{-1}$, K_{cat_2} = 5000 $\mu M^{-1}min^{-1}$, K_{cat_3} = 5000 $\mu M^{-1}min^{-1}$, K_{cat_4} = 500 $\mu M^{-1}min^{-1}$, K_{cat_5} = 144 $\mu M^{-1}min^{-1}$, K_{cat_6} = 500 $\mu M^{-1}min^{-1}$, K_{m_1} = 0.001 μM, K_{m_2} = 90 μM, K_{m_3} = 11 μM, K_{m_4} = 136.88 μM, K_{m_5} = 90 μM, K_{m_6} = 90 μM, d_{Ras} = 0.05 min^{-1}, d_{Ras} = 0.05 min^{-1}, d_{Raf} = 0.5 min^{-1}, d_{ppMEK} = 0.9 min^{-1}, d_{ppERK} = 0.42 min^{-1}, d_{ppMEK3} = 0.5 min^{-1}, d_{ppp38} = 0.9 min^{-1}, K_{i_erk} = 0.1 μM, K_{i_raf} = 0.2 μM, K_{i_p38} = 0.1 μM, Ras_t = 0.35 μM, Raf_t = 0.12 μM, MEK_t = 0.36 μM, ERK_t = 0.7 μM, $MEK3_t$ = 0.36 μM, $p38_t$ = 0.7 μM. The negative feedback mediated via JNK/p38 was increased by setting K_{i_p38} = 0.001 μM in order to simulate the effect of knocking down JNK/p38 DUSPs.

Supplemental figure 6. Influence of EGF, PDBu and DUSP16 siRNA on localization of ppERK1/2 and ERK1/2. HeLa cells were transfected with control siRNA or with siRNAs targeting DUSP16. They were then kept in medium with 0.1% FCS for 16 hours prior to stimulation (with 10 nM EGF or 1 μM PDBu for 0 min (ctrl.) or 10 min (EGF and PDBu). They were then fixed, stained (with DAPI, or for ppERK1/2 or ERK1/2) and imaged as described in the Materials and Methods. Each matched group of 3 images (DAPI, ppERK1/2 and ERK1/2) shows representative cells in approximately 1% of the entire area imaged for generation of the data shown in figure 6. For each fluorophore image acquisition times and contrast settings are identical and the scale bar is approximately 10 μm. Note that EGF and PDBu both caused pronounced increases in ppERK1/2 staining, whereas DUSP16 siRNA had no such effect, and that EGF, PDBu and DUSP16 siRNA all caused redistribution of ERK1/2 from the cytoplasm to the nucleus.

Supplemental figure 7. Frequency distribution plots for effects of PDBu and DUSP16 siRNA on ppERK1/2 and N:C ERK1/2. HeLa cells were transfected with control siRNA or with siRNAs targeting DUSP16. They were then cultured for 16 hours prior to stimulation (with 1 μM PDBu for 0 min (ctrl.) or 10 min (PDBu), fixation, staining and imaging as described in the Materials and Methods. Frequency distribution plots were generated from the individual cell measures of whole cell ppERK1/2 (upper panel) or N:C ERK1/2 (lower panel). Each trace was derived from image analysis of 6000-12000 individual cells and was used to define narrow ppERK1/2 bins (8-120 and 680-720 AFU, blocks in upper panel) that approximate the mean values for control and stimulated cells. These bins were then used to define cell subpopulations (matched for ppERK1/2 levels) that were used to generate the data in figure 6B.

$$\frac{d[Ras]}{dt} = \frac{K_{cat_1} s(t)(Ras_t - [Ras])}{\left(K_{m_1} + (Ras_t - [Ras])\right)\left(1 + \left(\frac{[ppERK]}{K_{i_{erk}}}\right)^{3.2}\right)} - d_{Ras}[Ras] \quad (1)$$

$$\frac{d[Raf]}{dt} = \frac{K_{cat_2} [Ras](Raf_t - [Raf])}{\left(K_{m_2} + (Raf_t - [Raf])\right)\left(1 + \left(\frac{[ppp38]}{K_{i_{p38}}}\right)^{3.2}\right)} - d_{Raf}[Raf] \quad (2)$$

$$\frac{d[ppMEK]}{dt} = \frac{K_{cat_3} [Raf](MEK_t - [ppMEK])}{\left(K_{m_3} + (MEK_t - [ppMEK])\right)\left(1 + \left(\frac{[ppERK]}{K_{i_{raf}}}\right)^{3.2}\right)} - d_{ppMEK}[ppMEK] \quad (3)$$

$$\frac{d[ppERK]}{dt} = \frac{K_{cat_4} [ppMEK](ERK_t - [ppERK])}{\left(K_{m_4} + (ERK_t - [ppERK])\right)} - d_{ppERK}[ppERK] \quad (4)$$

$$\frac{d[ppMEK3]}{dt} = \frac{K_{cat_5} s(t)(MEK3_t - [ppMEK3])}{\left(K_{m_5} + (MEK3_t - [ppMEK3])\right)} - d_{ppMEK3}[ppMEK3] \quad (5)$$

$$\frac{d[ppp38]}{dt} = \frac{K_{cat_6} [ppMEK3](p38_t - [ppp38])}{\left(K_{m_6} + (p38_t - [ppp38])\right)} - d_{ppp38}[ppp38], \quad (6)$$

Supplemental figure 1

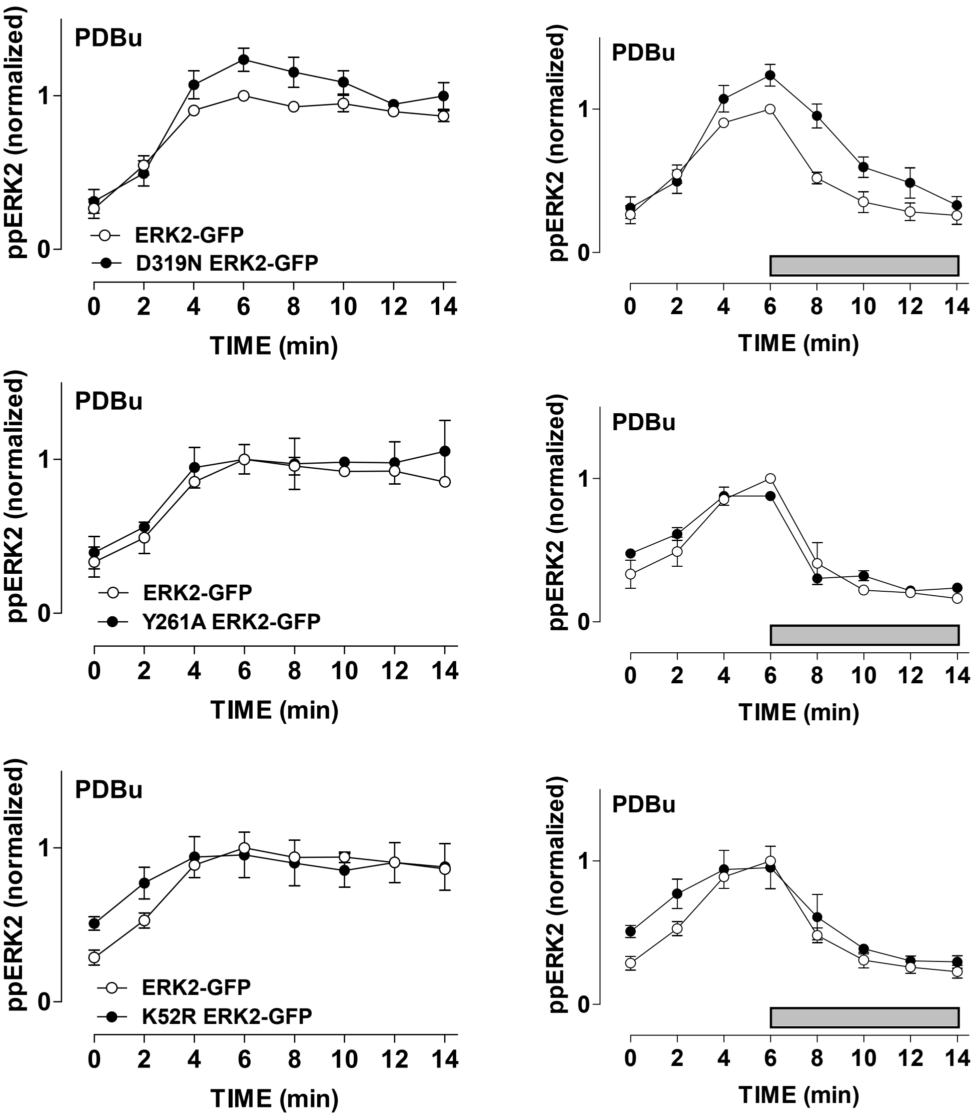

Supplemental figure 2

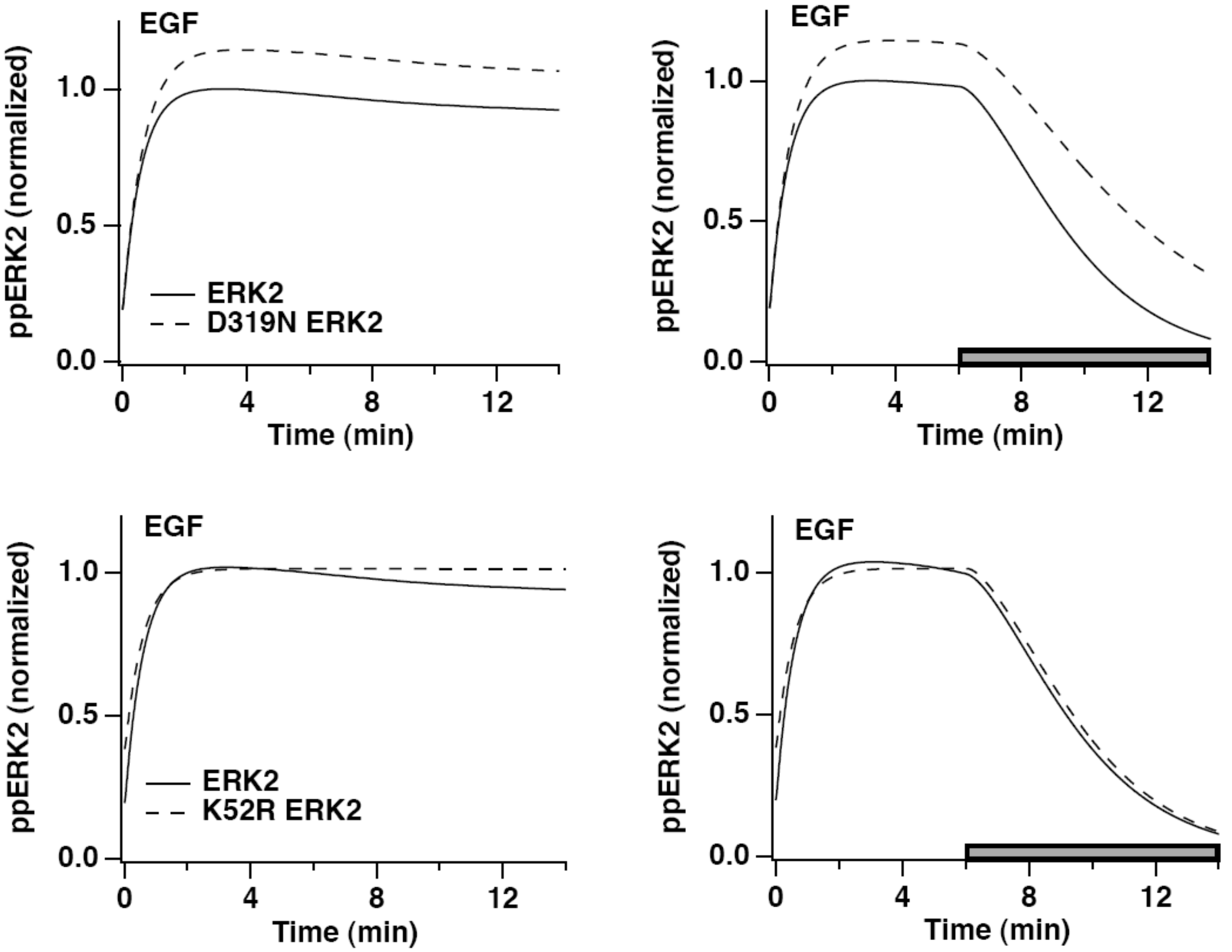

Supplemental figure 3.

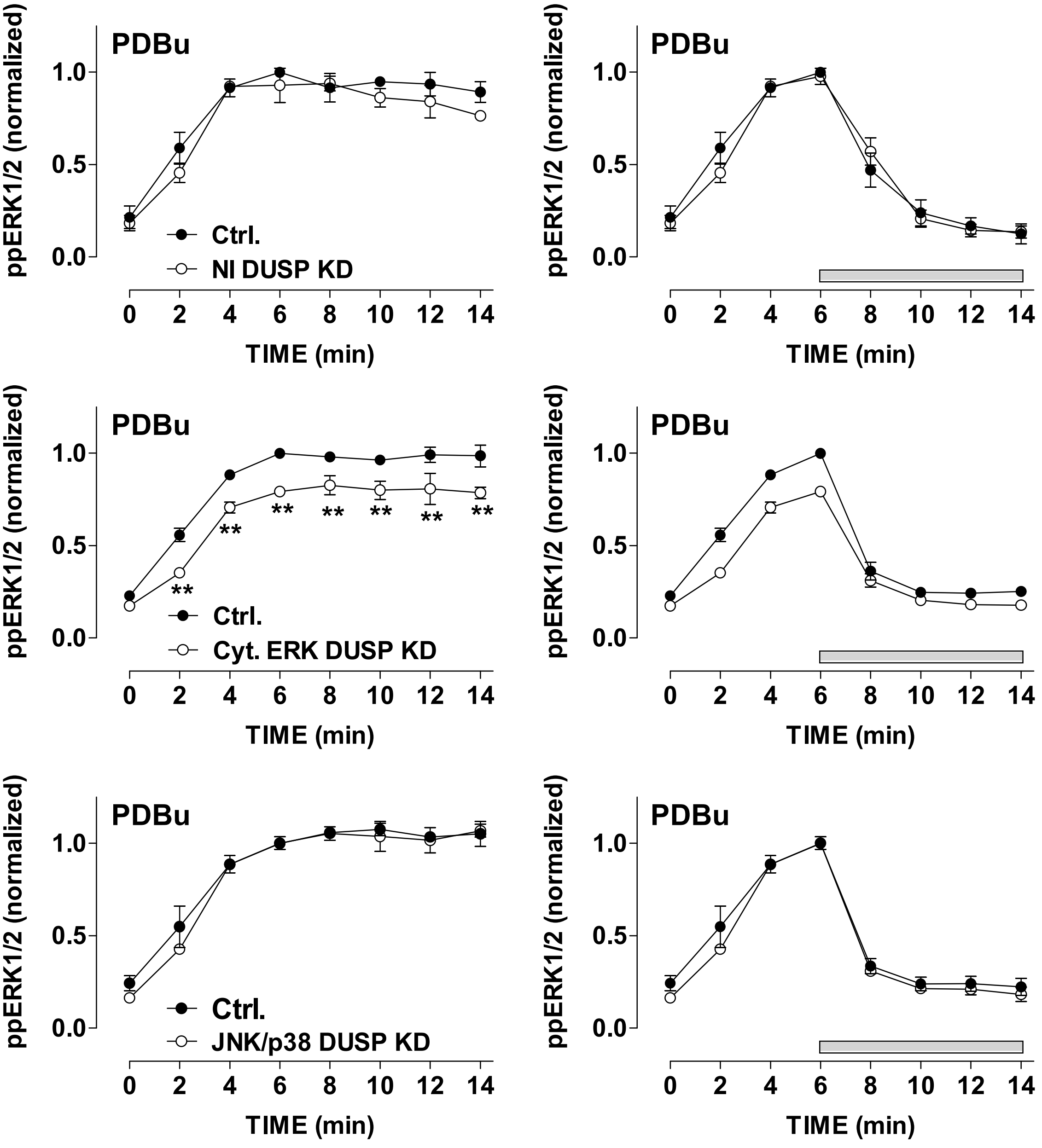

Supplemental figure 4

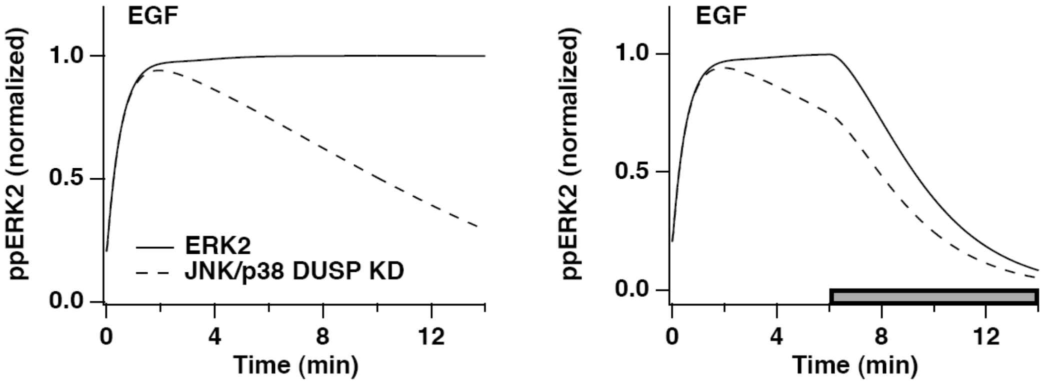

Supplemental figure 5.

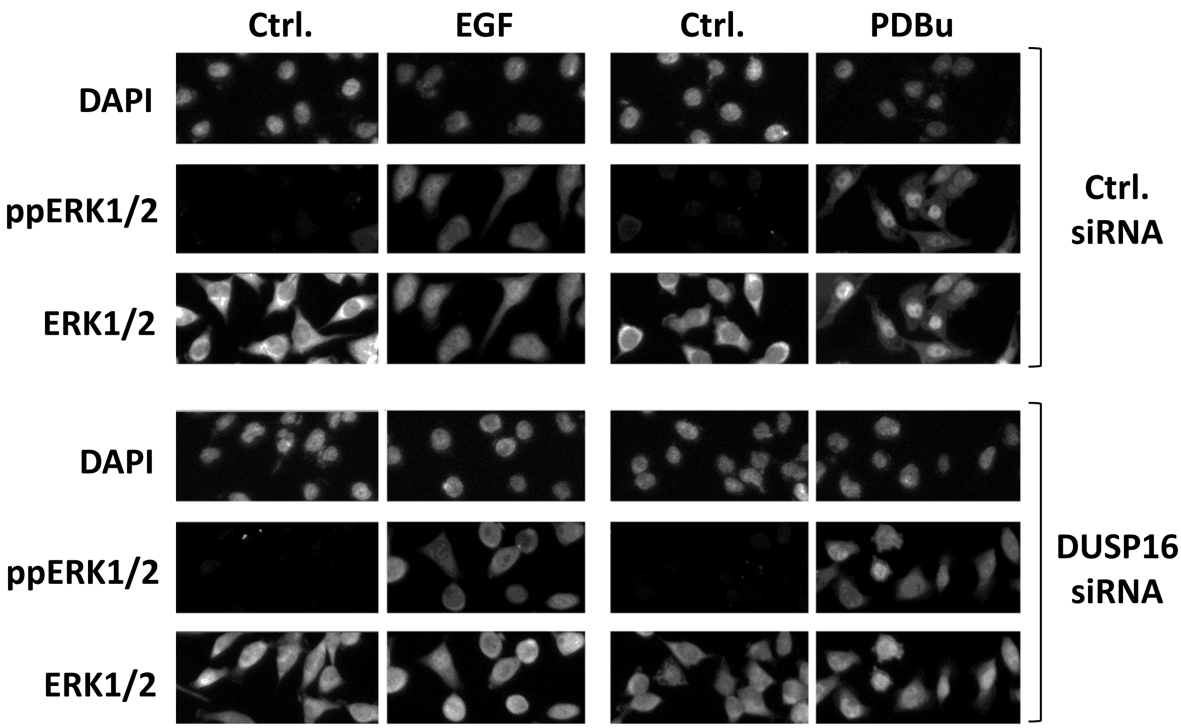

Supplemental figure 6.

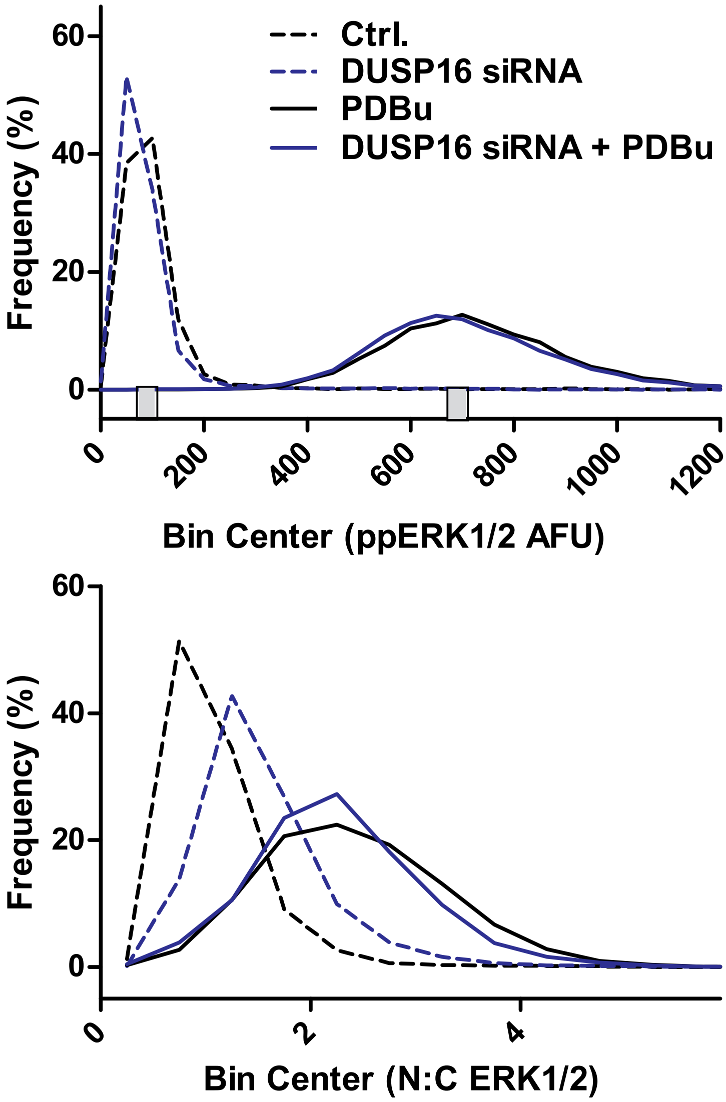

Supplemental figure 7
